# A prospective, double-blind, randomized, controlled clinical trial comparing standard wound care with adjunctive hyperbaric oxygen therapy (HBOT) to standard wound care only for the treatment of chronic, non-healing ulcers of the lower limb in patients with diabetes mellitus: a study protocol

**DOI:** 10.1186/1745-6215-12-69

**Published:** 2011-03-07

**Authors:** Daria O'Reilly, Ron Linden, Ludwik Fedorko, Jean-Eric Tarride, Wilhelmine Giffening Jones, James M Bowen, Ron Goeree

**Affiliations:** 1 Programs for Assessment of Technology in Health (PATH) Research Institute, St. Joseph's Healthcare Hamilton, 25 Main St. W., Suite 2000, Hamilton, Ontario, L8P 1H1, Canada; 2 Department of Clinical Epidemiology & Biostatistics, Faculty of Health Sciences, McMaster University, Hamilton, Ontario, Canada; 3 Judy Dan Research and Treatment Centre, 555 Finch Avenue West, 2nd Floor, Toronto, Ontario, M2R 1N5, Canada; 4 University Health Network, Department of Anaesthesiology, Toronto General Hospital, 200 Elizabeth Street, Toronto, Ontario, M5G 2C4, Canada

## Abstract

**Background:**

It has been suggested that the use of adjunctive hyperbaric oxygen therapy improves the healing of diabetic foot ulcers, and decreases the risk of lower extremity amputations. A limited number of studies have used a double blind approach to evaluate the efficacy of hyperbaric oxygen therapy in the treatment of diabetic ulcers. The primary aim of this study is to assess the efficacy of hyperbaric oxygen therapy plus standard wound care compared with standard wound care alone in preventing the need for major amputation in patients with diabetes mellitus and chronic ulcers of the lower limb.

**Methods/Design:**

One hundred and eighteen (59 patients per arm) patients with non-healing diabetic ulcers of the lower limb, referred to the Judy Dan Research and Treatment Centre are being recruited if they are at least 18 years of age, have either Type 1 or 2 diabetes with a Wagner grading of foot lesions 2, 3 or 4 on lower limb not healing for at least 4 weeks. Patients receive hyperbaric oxygen therapy every day for 6 weeks during the treatment phase and are provided ongoing wound care and weekly assessments. Patients are required to return to the study centre every week for an additional 6 weeks of follow-up for wound evaluation and management. The primary outcome is freedom from having, or meeting the criteria for, a major amputation (below knee amputation, or metatarsal level) up to 12 weeks after randomization. The decision to amputate is made by a vascular surgeon. Other outcomes include wound healing, effectiveness, safety, healthcare resource utilization, quality of life, and cost-effectiveness. The study will run for a total of about 3 years.

**Discussion:**

The results of this study will provide detailed information on the efficacy of hyperbaric oxygen therapy for the treatment of non-healing ulcers of the lower limb. This will be the first double-blind randomized controlled trial for this health technology which evaluates the efficacy of hyperbaric oxygen therapy in prevention of amputations in diabetic patients.

**Trial registration:**

ClinicalTrials.gov Identifier: NCT00621608

## Background

Non-healing foot ulcers and their sequelae are a major source of morbidity and resource use for patients with diabetes mellitus [1-4]. Peripheral neuropathy, peripheral vascular disease, and poor glycemic control, in conjunction with minor foot trauma, increase the likelihood that patients with diabetes will develop foot ulcers. Because of the neuropathy, a foot injury and subsequent infection cannot be felt and since circulation is also affected, wound healing is compromised and causes the original ulcer to become chronic and may eventually require amputation [5-7]. For those patients with ulcers, excess healthcare costs are substantial. Ramsey et al [8] found that the attributable cost for a 40- to 65-year-old male with a new foot ulcer was $27,987 USD for the 2 years after diagnosis. Not surprisingly, quality of life is significantly reduced in patients with ulcers and after major amputations [9].

In patients with diabetes, it is estimated that the annual incidence of foot ulcers varies from 1.2 to 3.0% [8,10] and the rate of lower extremity amputation (LEA) has been measured to range between 6% and 23.5% [11]. Major LEAs are amputations of the leg above or below the knee, whereas minor LEAs involve amputation of the toes or the forefoot [12]. The incidence of major amputation in a study of 8,905 patients with diabetes in the United States was found to be 0.9% [8]. Additionally, a significant proportion of these amputees undergo further amputations of the same or other limb [8,11,13,14].

The standard of care for treating diabetic foot ulcers includes the maintenance of optimal blood glucose levels; use of debridement, antibacterials, and dressings; administration of antibiotics to control infection; adequate nutrition; pressure relief in the areas of the foot that are most subject to weight bearing; and amputation. There has also been increasing interest in the use of hyperbaric oxygen therapy (HBOT) as an adjunctive treatment for diabetic ulcers. It has been suggested that the use of adjunctive HBOT will improve the healing of diabetic lower leg ulcers, and decrease the risk of LEA [15-20].

HBOT is an established technology that has been in use for more than 40 years. For wound healing treatment, a person is placed in a compression chamber under pressure greater than one atmosphere absolute (ATA) of 100% oxygen. The pressure increases the level of oxygen dissolved in the blood plasma affecting the immune system, wound healing, and vascular tone [21]. Treatment regimens vary from 90-120 minutes once or twice daily for approximately 30 sessions [22]. Complications associated with HBOT are infrequent but may include claustrophobia; ear, sinus or lung damage due to the pressure; temporary worsening of short sightedness, rarely is permanent; and oxygen poisoning [23]. Careful monitoring during the treatment sessions and follow-up by a trained health care provider is recommended.

There is evidence to support that adjunctive HBOT is more effective in the treatment of chronic diabetic foot ulcers than standard care alone. However, this evidence of efficacy for chronic diabetic foot ulcers is limited. In particular, several, very small, prospective, randomized, controlled trials can be identified in the published literature to date [16,17,19]. The first trial [17] randomized 30 patients, the second randomized 68 patients [16]. Only two double blind placebo controlled trials have ever been reported including a trial conducted in 2003 which randomized just 16 patients [19], while the more recent largest trial to date successfully randomized and completed 37 patients in each arm [24]. In this latest study, the primary outcome was healing of the index wound, however, the healing rate in the control group in this study was very low at 29%. All trials demonstrated that more wounds healed in HBOT-treated patients compared to the control group. Two of the three studies [16,17] demonstrated a reduction of major LEA in the HBOT group, while the other study showed no difference between the groups. In addition, successes of adjunct HBOT for the treatment of diabetic foot ulcers have been reported in a few small case series and retrospective or prospective cohorts [15,18,25-28]. However, many of these originated from one clinical centre [16,18,27,28].

Using this limited evidence, several health technology assessments and systematic literature reviews have been conducted that suggested that adjunctive HBOT for diabetic foot ulcers is more effective than standard care alone [29-37]. At the same time, all reports recommend that good quality studies are required to confirm the comparative benefits of the technology for chronic diabetic foot ulcers in order to help clinicians and policy-makers decide whether HBOT should be more widely utilized [33,34,37].

Currently in Ontario, HBOT is an accepted treatment for chronic diabetic ulcers and physicians who provide this service are reimbursed under the current Ontario Health Insurance Plan (OHIP) payment schedule. However there are only a few facilities which provide comprehensive wound care and adjunctive HBOT.

A recent review of the HBOT literature concluded that the quality of the evidence assessing the efficacy of HBOT as an adjunct to standard therapy for people with non-healing diabetic foot ulcers is low, and the results are inconsistent [32]. Thus, the goal of the current study is to conduct a well-designed, double-blind randomized controlled trial that would provide quality efficacy data on the use of adjunctive HBOT for the prevention of major limb amputations for patients with moderate and severe chronic diabetic foot ulcers. To better assess the place of HBOT in modern wound care, the best practice principles of treating chronic diabetic foot ulcers are applied in both control and treatment arms.

This study was initiated based on a recommendation by the Ontario Health Technology Advisory Committee that stated that a "well designed, adequately powered, clinical trial be undertaken to provide evidence on which to make a definitive decision on the effectiveness of HBOT in the treatment of non-healing diabetic foot ulcers".

### Objectives

#### Primary Objective

This study is intended to assess the efficacy of HBOT plus standard wound care compared with standard wound care alone in preventing the need for major amputation in patients with diabetes (Type 1 or 2) with moderate to severe chronic wounds of lower limbs (Wagner scale 2 to 4) [38].

#### Secondary Objectives

Further objectives of the study are to determine if HBOT, in combination with standard wound care, significantly improves the healing of chronic ulcers compared with standard wound care alone through the assessment of wound measurements and wound closure. The safety, amount of health care resource utilization, health-related quality of life (HRQL), and cost-effectiveness of HBOT will also be measured.

### Hypothesis

HBOT plus standard wound care is more effective than standard wound care alone at preventing the need for major amputation (metatarsal and up) in patients with diabetes with moderate to severe chronic wounds of lower limbs.

## Methods/Design

### Design

**Figure 1:**
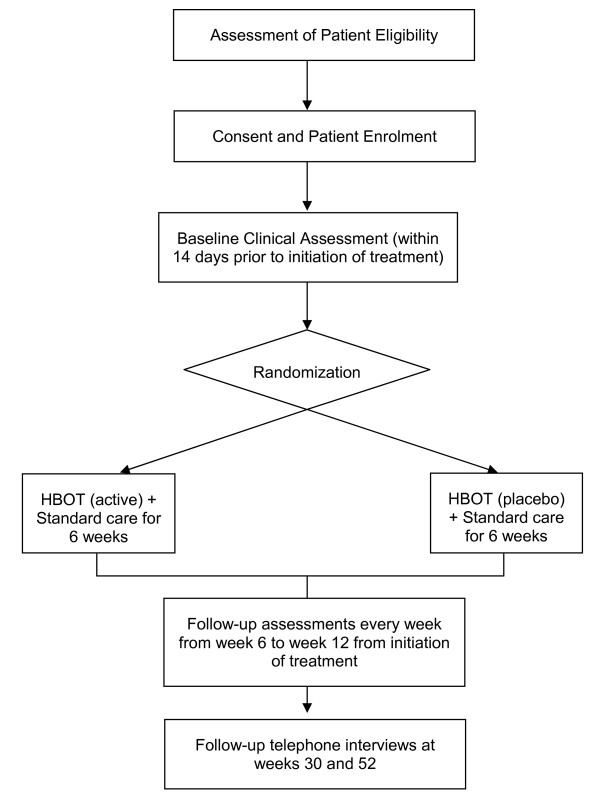
**Study Schema**.

Patients are randomised either to receive standard wound care alone or HBOT in combination with standard wound care for the treatment of chronic lower limb ulcers in patients with diabetes (Figure 1).

### Setting and participants

This study is being conducted at the Judy Dan Wound Care and Research Centre an University Health Network Research affiliated wound care facility. Patients with non-healing diabetic lower limb ulcers are referred by physicians and also identified through a number of wound care clinics in the Greater Toronto Area (GTA) as well as from regional Community Care Access Centres.

### Inclusion Criteria

1) Age ≥ 18 years; 2) Type 1 or 2 Diabetes Mellitus; and 3) Wagner grading [38] of foot lesions 2,3 or 4 on lower limb not healing for at least 4 weeks.

### Exclusion Criteria

1) Impending urgent amputation due to ongoing or exacerbated infection; 2) exposed calcaneus bone with no prospect of weight bearing potential even if defect has been healed; 3) any of the following medical conditions which preclude safe treatment in a monoplace chamber: clinical depression; severe dementia; claustrophobia; seizure disorder; active asthma; severe chronic obstructive pulmonary disease; previous thoracic surgery; previous spontaneous or trauma induced pneumothorax; history of severe congestive heart failure with left ventricular ejection fraction less than 20%; unstable angina; chronic sinusitis; chronic or acute otitis media or major ear drum trauma; severe kyphoscoliosis; severe arthritis; or morbid obesity; 4) history of chemotherapy with use of Bleomycin; 5) participation in another investigative drug or device trial currently or within the last 30 days; 6) current candidates for vascular surgery, angioplasty or stenting; 7) major large vessel disease; 8) undergone vascular surgery or angioplasty within the last 3 months; 9) women who are currently pregnant or are breast feeding or women of childbearing potential who are not currently taking adequate birth control.

### Sample Size

A comprehensive search and review of the electronic medical literature databases was conducted to identify randomized controlled trials of HBOT versus standard treatment to collect data to calculate the sample size required for the current study. Sample size calculations were to be based on evidence from published randomized controlled trials comparing the efficacy, in terms of rates of major amputation, of HBOT versus standard treatment for patients with various wound classifications using the Wagner's Ulcer Classification Grade [38].

Of the 348 citations obtained, only one study [16,19] reported the rates of major amputation for Wagner's classifications necessary for the calculation of sample size for this study given that it was decided a priori that patients with Wagner Grades of 2,3 or 4 would be recruited into the study. Based on these data, the expected event rate in the standard care group was 39.29% and 11.54% in the HBOT group and using a two-sided test for equality.

Using these data, it was estimated that a total of 94 patients were needed for this study (47 patients per treatment group). However, due to the fact that subjects might leave the study for a variety of reasons (e.g. loss to follow-up, non-compliance with treatment, or death), the sample size was adjusted to accommodate for this. It was assumed that there was a likely dropout rate of 20%. This assumption would require a total of 118 subjects, or 59 subjects in each treatment group (i.e., 94/(1-0.20) = 118).

### Recruitment of patients

Two streams of referrals are utilized:

1. Patients referred by primary care physicians undergo full assessment, including assessment by the participating vascular surgeon and a foot care specialist to determine best treatment options for the patient. Once it has been determined that they are not candidates for surgical/angioplasty/stenting and they fulfill inclusion criteria they are asked to participate in the trial.

2. Patients referred by specialists, most commonly vascular and plastic surgeons, usually have already undergone detailed assessment and their wounds deemed not amenable by interventional techniques. Previous test results are forwarded to the participating vascular surgeon to examine whether a sufficient level of assessment has been performed, and whether inclusion/exclusion criteria are met, the patients are then asked to participate in the study.

### Randomisation and Blinding

Patients are randomized using a computerized block randomization schedule with a multiple block size of four. The unblinded HBOT technician obtains the treatment allocation through an internet-based automated randomization system. Researchers and patients are blinded to treatment allocation; the only unblinded individual is the technician responsible for controlling the hyperbaric oxygen chamber.

### Interventions

#### Hyperbaric oxygen therapy

Within 2 weeks of enrolment, each study participant is placed into the hyperbaric chamber, but only those patients allocated to active HBOT receive 90 minutes of oxygen at 2.4 ATA with the patients breathing 100% oxygen when inside the chamber. Those patients randomized to placebo will be compressed on air to 0.3 ATA (10 feet) and kept at that level. The patient will remain in the chamber for the remainder of the placebo treatment breathing normally. At the end of the treatment, after a short period of enhanced ventilation (to simulate surfacing) the chamber will be opened. Patients enter the chambers 5 days per week for approximately 6 weeks for a total of 30 treatments. At the end of the 6 week treatment phase, patients enter a 6-week follow-up phase.

#### Dressing changes

Based on the characteristics of the wound, dressings will be changed as required (at a minimum of 2 dressing changes per week). Wound care is standardized throughout the entire study to several different dressing types (e.g. silver, simple gauze, and alginate, collagen/oxidized cellullose) dependent on the type of the wound (e.g. dry, wet, and intermediate). The very wet wounds receive daily dressing changes as required by standard of care. Clinical signs of infection are noted and antibiotic therapy initiated if required and swabs obtained. Referral for debridement of the wound occurs if necessary.

### Data collection

Data to evaluate the efficacy of HBOT are collected at screening, baseline, and weekly throughout the 6-week treatment phase, weekly throughout the 6-week follow-up phase at 30 and 52 weeks. At the end of the 6-week follow-up phase (i.e. week 12), the patient is sent to the participating vascular surgeon for an amputation evaluation. If the patient's wound has not healed, the wound care physician, in consultation with the vascular surgeon will then decide if further HBOT or other treatment is indicated. Once all study data for each patient are received, the randomization assignment may be revealed. Data are collected via interview, self administrated questionnaire, physical and biological measurements. Data collection instruments and the study timeline are summarized in Table 1. All instruments used have been previously validated.

**Table 1:** Schedule of study assessments and evaluations

			Treatment Phase		Follow-up Phase		Long-Term Follow-up	
			
Clinical Assessments, Testing and Investigations	Screening and Randomization	Baseline	Weeks 1 to 6	End of Treatment	Weeks 6 to 12	End of Follow-up	Week 30	Week 52
Suitability for HBOT	X							

Electrocardiogram	O							

Pulmonary Function Testing	O	X						

Audiogram	O							

Chest X-ray	O							

X-ray of affected limb	O	X						

Nuclear bone scan	O							

Vascular Exam	X	O	O	O	O	X		

History & Physical		X						

Demographics		X						

Treatment Details		X	X					

Wound Classifications (Wagner, Bates)	X	X	X		X			

Wound Measurements		X	X		X			

Wound Characteristics		X	X		X			

Digital Photograph of wound		X	X		X			

Dressing Changes	X	X	X		O	O		

Concomitant Meds		X	X		X			

Assess for Infection	X	X	X		X			

Mobility Status		X	X		X			

Adverse Events		X	X		X		X	X

Outcome Assessment	X	X	X		X			

Unblinding		O	O	O	O	O		

Laboratories	X	X		X		X		

Resource Utilization		X	X		X		X	X

**Quality of Life**:								

SF-36		X		X		X		

EQ-5D		X		X		X	X	X

DFS-SF		X		X		X		

x = required o = optional

SF-36: Short-Form 36; EQ-5D: EuroQoL 5D; DFS-SF: Diabetic Foot Ulcer Scale - Short Form

### Primary Outcome

The primary outcome in this study is freedom from having, or meeting the criteria for, a major amputation (below knee amputation, or metatarsal level) up to 12 weeks after initiation of treatment. The decision to amputate is made by a vascular surgeon and by meeting any of the following criteria:

1. Persistent deep infection involving bone and tendons (antibiotics required, hospitalization required, pathogen involved);

2. Ongoing risk of severe systemic infection related to the wound;

3. Inability to bear weight on the affected limb;

4. Pain causing significant disability.

### Secondary Outcomes

#### 1) Wound healing

Measures of wound healing include difference in wound measurements (i.e. depth, length, width and extent of surface area) using: a digital photograph; reduction in Wagner Classification Score; Bates-Jensen Wound Assessment score; proportion of wounds closed on or before 12 weeks; and time to healing (days).

##### Digital Photographs for Healing Assessment

Using a digital camera with PictZar^® ^CDM Software, digital images of the wounds are captured and downloaded to a computer. Once downloaded to a computer, PictZar^® ^CDM Software is used to make various surface measurements on the wound photograph (i.e. depth, length, width, area, circumference and volume). The software provides a method of calibrating digital images based on a calibration strip placed on the skin adjacent to the wound. Photographs are used for comparison to assess the degree of ulceration.

##### Wagner Scale

The foot ulcer classification system used in this study was described and popularized by Wagner [38]. In the Wagner system, the natural history of dysvascular foot breakdown is divided into six grades ranging from Grade 0 (preulcer) to Grade 5 (amputation required). The system is similar to an ordinal scale denoting ranked order, allowing for nonparametric data analysis. Grade is determined based on depth of the skin lesion and the presence or absence of infection and gangrene.

##### Bates-Jensen Wound Assessment Tool

The Bates-Jensen Wound Assessment Tool (Barbara Bates-Jensen ^© ^2001) [39], is a validated wound assessment tool that evaluates 13 wound characteristics with each item scored on a 1-5 scale. A total score is obtained by adding all individual scores and the results are plotted on the Wound Status Continuum. Higher total scores indicate a more severe wound status.

#### 2) Effectiveness

Maintenance on therapy (discontinuation rates) and secondary prevention interventions (confounding variables that may influence primary outcome such as diabetes control) are collected.

#### 3) Safety

Major morbidity (e.g. infection requiring hospitalization, acute coronary syndrome, renal failure); wound interventions during study (debridement, surgery); complications related to HBOT (seizure, pulmonary syndromes, vision disturbance, middle ear or sinus problems); and all cause mortality are recorded on the case report forms.

#### 4) Healthcare resource utilization

All healthcare resources used by study patients during the course of the study are documented (e.g. wound dressing materials, healthcare provider visits; inpatient hospital admissions; complex continuing care/rehabilitation; drug therapy; mobility assistive devices, etc.).

#### 5) Quality of life

Diabetic foot ulcers are associated with pain, discomfort and immobility, which could lead to anxiety, depression, and isolation. As a result, one of the secondary objectives of this study is to measure any changes in HRQL in the two treatment groups. Three different instruments are utilized: 1) a generic quality of life instrument - Short-Form 36; 2) a preference-based or utility measure - EuroQoL 5D (EQ-5D); and 3) a disease-specific questionnaire - Diabetic Foot Ulcer Scale - Short Form. Self-assessment will be performed at baseline, 6 weeks, and 12 weeks in order to monitor the progress or decline of the patient through their own interpretation. Only the EQ-5D will be administered at weeks 30 and 52.

#### 6) Cost effectiveness of HBOT

The final objective of this study is to assign cost values to the healthcare resource utilization data in order to determine the incremental cost per amputation avoided and the incremental cost per quality-adjusted life-year (QALY) gained associated with the treatments.

## Early Discontinuation

If a patient's wound heals during the treatment phase, they discontinue the HBOT treatment visits but continue their weekly visits until week 12. Dressing changes and prevention care plans are put in place and wound evaluation visits include the documentation of healthcare resource utilization, quality of life measurements, and any relevant clinical information.

Alternatively, if amputation criteria are met during the treatment phase, the outcome is recorded and efforts made to maintain wound care as per protocol until amputation procedure can be done or if systemic infection or ascending infection then immediate referral to surgery. Healthcare resource utilization is collected weekly and quality of life is collected for these patients as per protocol (i.e. 6 and 12 weeks).

If a patient discontinues the assigned treatment due to inability to tolerate or unwilling to comply with therapy but is still willing to remain in the study, the patient is provided standard wound care. Wound evaluation visits are completed when possible.

If a patient needs to temporarily discontinue treatment due to a medical illness, infection in the wound where HBOT cannot be continued, or complications of the wound, this is considered a temporary interruption. Once the patient has recovered, they may begin a new HBOT treatment regimen. All patients who have temporarily had their treatment interrupted will be followed for all regular clinical wound evaluation (Table 1).

## Statistical Analysis

### Data Analysis

Baseline variables will be evaluated for balance between the two groups using the Student's t-test for unpaired data for the comparison of continuous variables and the Pearson's chi-squared test to compare categorical variables.

For the primary outcome of rate of, or criteria for, major amputation, the chi-squared test will be used. Additionally, a relative risk will be estimated for the major amputation rates in each arm and multivariable logistic regression analysis of major amputations will be performed using a number of covariates.

All test instruments for the various secondary outcomes will be scored according to the recommendations for the particular tests, with missing values handled in an appropriate fashion (e.g., prorated sum, average) and corrections for repeated measures will be applied where appropriate. Scores for any patients who die prior to the end of study from causes directly related to their wound (e.g. sepsis) are considered to have required an amputation. Patients who die from other causes are eliminated from further analyses due to lack of follow-up data but the outcome recorded. When patients are unwilling to complete a follow-up visit or are lost to follow-up we will use chi-squared analysis to determine whether there is imbalance between the treatment groups with respect to loss of follow-up.

The extent of healing of the ulcers will also be assessed by measuring the ulcer surface area from the digital photographs. Since it is a continuous variable, data will be tested for normality and analyzed as appropriate with t-test or non-parametric test if required.

All analyses will be based on the "intention-to-treat" principle. Per-protocol analyses will also be conducted.

### Subgroup Analysis

There are no planned subgroup analyses anticipated. Only primary and secondary outcome measures will be analyzed. However, all of the variables which are found to be important predictors of amputation and adverse outcomes, will be used for stratifications for post-hoc analyses.

### Health Economic Evaluation

An economic evaluation will be conducted in order to assess the cost-effectiveness of HBOT plus standard wound care compared to wound care alone. The analysis will be based upon patient-level outcome and resource use data from the trial. Two cost-effectiveness outcomes will be measured: 1) cost per major amputation avoided; and 2) the cost per QALY gained. Modeling techniques will be used to extend the time horizon beyond the duration of the trial. A third-party payer perspective (i.e. Ontario Ministry of Health) will be taken in the evaluation.

Healthcare resource utilization over the duration of the trial is measured for each patient. A number of different cost components are being considered: HBOT treatment; follow-up wound care visits; inpatient admissions; general practitioner visits; specialist visits; other allied healthcare professional visits (e.g. chiropodists, physiotherapists); and mobility assistive devices (e.g. wheelchairs, walkers). The cost per HBOT treatment will be based upon the Judy Dan Wound Care Centre operating budget. The cost for nurse wound care will be based upon the average hourly nursing wage in Ontario. For patients in the active HBOT plus standard care arm, treatment costs will include both HBOT and wound care nurse costs. For patients in the standard wound care arm, treatment costs will not include HBOT costs.

Follow-up resource use data will be assessed at the completion of HBOT treatment (week 6), weeks 12, 30 and 52. Appropriate unit costs will be applied to the resource use data to estimate follow-up costs for each patient. Unit costs will be derived from a number of sources including the Ontario Case Costing Initiative database and the OHIP Schedule of Benefits.

QALYs for each treatment arm will be based upon utilities measured over the duration of the trial. Uncertainty of cost-effectiveness results will be estimated using non-parametric bootstrap techniques and presented using cost-effectiveness acceptability curves.

## Study Organization

### Steering Committee

The Steering Committee, which consists of the clinical and methodological investigators involved with the study, is responsible for the overall design of the study and ensures that the execution and management are of the highest quality. Committee members from the PATH Research Institute have extensive knowledge in clinical trial design, data analysis and economic evaluation techniques. The clinical investigators bring extensive knowledge in HBOT wound treatment. The Steering Committee corresponds on a regular basis to review the conduct of the study, specifically data related to: rates of accrual, major protocol deviations and adverse events.

### Programs for Assessment of Technology in Health (PATH) Research Institute

The PATH Research Institute, based out of St. Joseph's Healthcare Hamilton, located in Hamilton, Ontario, is responsible for the overall coordination of the study, including protocol development and finalization, design of case report forms, operations manuals, study materials and data management (e.g. data collection, validation, data clarifications). During the study, the PATH Research Institute is responsible for monitoring the study execution, particularly with respect to the methodological aspects, ensuring adherence to the study protocol by the clinical centre and study personnel involved, preparation of summary information and reports.

The PATH Research Institute is responsible for the final analysis of the dataset and, in consultation with the investigators, will complete a final report regarding the use of HBOT to the Ontario Ministry of Health and Long-term Care. This study is part of a larger program of research by the PATH Research Institute of conducting "field evaluations' with an overall goal to collect necessary information of new emerging health technologies in Ontario.

## Deviations From Protocol

Patients must meet the inclusion and exclusion criteria to be enrolled in the study. If, after enrolment, it is noted that the patient does not meet the inclusion and/or exclusion criteria, the information is recorded on the enrolment case report forms. A protocol deviation report is prepared and kept on file at the clinical centre as well as on file at the coordinating centre. In addition, any deviations from the protocol for the remainder of the study are documented on the case report forms.

### Ethical Considerations

This study is being conducted in compliance with the Canadian Tri-Council Policy Statement: Ethical Conduct for Research Involving Humans (TCPS), Health Canada Food and Drug Regulations, and the International Conference on Harmonization (ICH) Guidelines (E6) for Good Clinical Practice (GCP) and in agreement with the latest revision of the Declaration of Helsinki, Personal Health Information Protection Act privacy legislation and all applicable Canadian laws and regulations, as well as any local laws and regulations and all applicable guidelines. This protocol and any amendments have Research Ethics Board (REB) approval at St. Joseph's Healthcare Hamilton (the sponsoring site) (#: 08-3017) and the University Health Network in Toronto (# 07-0586-AE). The approval of the REB concerning the conduct of the study was made in writing to the investigator before commencement of the study. An investigator provides the REB with reports, updates and other information periodically, according to the regulatory requirements or Institution procedures.

### Subject Consent

The informed consent of a patient is obtained from all potential study participants using the REB-approved Patient Informed Consent form. The clinical investigator, or a person designated by the clinical investigator, and under the clinical investigator's responsibility, informs the potential study subject of all pertinent aspects of the study. All potential participants are informed of the study in a language and terms they are able to understand. Subjects are also informed that their medical care will not be affected should they choose not to participate. Prior to a subject's participation in the study, the Subject Information and Informed Consent form is signed, name printed and personally dated by the subject or the subject's legally acceptable representative, and by the person who conducted the informed consent discussion. A copy of the signed and dated Subject Information and Informed Consent form is provided to the subject. Documentation that the informed consent was signed and dated prior to any study procedures being performed is made at the time of the informed consent and appears in a source document at the clinical centre, such as the subject's medical record.

### Adverse Events

This study is being conducted according to the ICH Good Clinical Practice Guidelines. Adverse events and serious adverse events information are documented.

### Confidentiality

All records identifying the study subject are kept confidential and, to the extent permitted by the applicable laws and/or regulations, will not be made publicly available. The patient's name appears in the patient consent form and the contact information sheet which collects relevant information that the clinical centre will need to arrange follow-up visits. This information is recorded locally at the clinical centre only and will never be received at the coordinating centre and therefore no patient identification information is recorded in the study database. At the time of enrolment, each patient is assigned a unique study identification number. Only the subject identification number and subject initials are recorded on the case report forms. If the subject name appears on any other documents (i.e. source documents), it must be obliterated before a copy of the document is sent to the coordinating centre. Study findings stored on a computer are protected in accordance with local data protection laws.

## Disscussion

This study has been designed to address some of the shortcomings of previous research, namely this study will be the largest and first double-blind randomised controlled trial to evaluate the efficacy of HBOT for the prevention of major amputations in diabetic patients with non-healing ulcers of the lower limb. The study uses a remote randomization system to protect concealment of allocation and proposes that the primary comparative analyses be conducted on an intention-to-treat basis in accordance with the 2010 CONSORT statement [40].

Participants are followed up for a 12-month period in order to capture the impact of HBOT treatment in both the immediate (i.e. 12 weeks) and longer term. This two-arm study, with 49 participants per group, should provide sufficient power to detect a meaningful difference in amputation. If successful, this intervention may lead to substantial and important changes in the management of diabetes patients at risk of amputation due to non-healing ulcers of the lower limb.

## Competing interests

The authors declare that they have no competing interests.

## Authors' contributions

DO'R, RL, LF and JB all made substantial contributions to the conception and design of the study. DO'R wrote the manuscript. JET, WGJ and RG provided critical review of the article and helped to draft the manuscript. All authors read and approved the final manuscript.

## References

[B1] Moss SE Klein R Klein BE Long-term incidence of lower-extremity amputations in a diabetic population Arch Fam Med 1996 5 7 391 8 10.1001/archfami.5.7.391 8664997

[B2] Bild DE Selby JV Sinnock P Browner WS Braveman P Showstack JA Lower-extremity amputation in people with diabetes Epidemiology and prevention. Diabetes Care 1989 12 1 24 31 10.2337/diacare.12.1.24 2714164

[B3] Humphrey AR Dowse GK Thoma K Zimmet PZ Diabetes and nontraumatic lower extremity amputations. Incidence, risk factors, and prevention--a 12-year follow-up study in Nauru Diabetes Care 1996 19 7 710 4 10.2337/diacare.19.7.710 8799624

[B4] Lehto S Ronnemaa T Pyorala K Laakso M Risk factors predicting lower extremity amputations in patients with NIDDM Diabetes Care 1996 19 6 607 12 10.2337/diacare.19.6.607 8725860

[B5] Brauchle M Funk JO Kind P Werner S Ultraviolet B and H2O2 are potent inducers of vascular endothelial growth factor expression in cultured keratinocytes J Biol Chem 1996 271 36 21793 7 10.1074/jbc.271.36.21793 8702976

[B6] Faglia E Mantero M Caminiti M Caravaggi C De GR Pritelli C Clerici G Fratino P De CP Dalla PL Mariani G Poli M Settembrini PG Sciangula L Morabito A Graziani L Extensive use of peripheral angioplasty, particularly infrapopliteal, in the treatment of ischaemic diabetic foot ulcers: clinical results of a multicentric study of 221 consecutive diabetic subjects J Intern Med 2002 252 3 225 32 10.1046/j.1365-2796.2002.01015.x 12270002

[B7] Faglia E Favales F Quarantiello A Calia P Clelia P Brambilla G Rampoldi A Morabito A Angiographic evaluation of peripheral arterial occlusive disease and its role as a prognostic determinant for major amputation in diabetic subjects with foot ulcers Diabetes Care 1998 21 4 625 30 10.2337/diacare.21.4.625 9571354

[B8] Ramsey SD Newton K Blough D McCulloch DK Sandhu N Reiber GE Wagner EH Incidence, outcomes, and cost of foot ulcers in patients with diabetes Diabetes Care 1999 22 3 382 7 10.2337/diacare.22.3.382 10097914

[B9] Valensi P Girod I Baron F Moreau-Defarges T Guillon P Quality of life and clinical correlates in patients with diabetic foot ulcers Diabetes Metab 2005 31 3 Pt 1 263 71 10.1016/S1262-3636(07)70193-3 16142017

[B10] Muller IS de Grauw WJ van Gerwen WH Bartelink ML van Den Hoogen HJ Rutten GE Foot ulceration and lower limb amputation in type 2 diabetic patients in dutch primary health care Diabetes Care 2002 25 3 570 4 10.2337/diacare.25.3.570 11874949

[B11] Faglia E Favales F Aldeghi A Calia P Quarantiello A Barbano P Puttini M Palmieri B Brambilla G Rampoldi A Mazzola E Valenti L Fattori G Rega V Cristalli A Oriani G Michael M Morabito A Change in major amputation rate in a center dedicated to diabetic foot care during the 1980s: prognostic determinants for major amputation Journal of Diabetes & its Complications 1998 12 2 96 102 10.1016/s1056-8727(97)98004-1 9559487

[B12] National Diabetes Education Program Foot health and diabetes: prevalence of foot symptoms and complications. National Diabetes Education ProgramBethesda (MD) 2004 National Insitutes of Health

[B13] Abbott CA Carrington AL Ashe H Bath S Every LC Griffiths J Hann AW Hussein A Jackson N Johnson KE Ryder CH Torkington R Van Ross ER Whalley AM Widdows P Williamson S Boulton AJ The North-West Diabetes Foot Care Study: incidence of, and risk factors for, new diabetic foot ulceration in a community-based patient cohort Diabet Med 2002 19 5 377 84 10.1046/j.1464-5491.2002.00698.x 12027925

[B14] Cianci P Petrone GJ Shapiro RL Ross J Lueders HW Economic considerations on the impact of adjunctive hyperbaric oxygen in potential amputees Proceedings of IXth International Symposium on Underwater and Hyperbaric Physiology 1987 Bethesda, MD: Undersea and Hyperbaric Medical Society 1075 9

[B15] Kalani M Jorneskog G Naderi N Lind F Brismar K Hyperbaric oxygen (HBO) therapy in treatment of diabetic foot ulcers. Long-term follow-up Journal of Diabetes & its Complications 2002 16 2 153 8 10.1016/s1056-8727(01)00182-9 12039398

[B16] Faglia E Favales F Aldeghi A Calia P Quarantiello A Oriani G Michael M Campagnoli P Morabito A Adjunctive systemic hyperbaric oxygen therapy in treatment of severe prevalently ischemic diabetic foot ulcer. A randomized study Diabetes Care 1996 19 12 1338 43 10.2337/diacare.19.12.1338 8941460

[B17] Doctor N Pandya S Supe A Hyperbaric oxygen therapy in diabetic foot J Postgrad Med 1992 38 3 112 4 111 1303408

[B18] Baroni G Porro T Faglia E Pizzi G Mastropasqua A Oriani G Pedesini G Favales F Hyperbaric oxygen in diabetic gangrene treatment Diabetes Care 1987 10 1 81 6 10.2337/diacare.10.1.81 3568965

[B19] Abidia A Laden G Kuhan G Johnson BF Wilkinson AR Renwick PM Masson EA McCollum PT The role of hyperbaric oxygen therapy in ischaemic diabetic lower extremity ulcers: a double-blind randomised-controlled trial Eur J Vasc Endovasc Surg 2003 25 6 513 8 10.1053/ejvs.2002.1911 12787692

[B20] Kessler L Bilbault P Ortega F Grasso C Passemard R Stephan D Pinget M Schneider F Hyperbaric oxygenation accelerates the healing rate of nonischemic chronic diabetic foot ulcers: a prospective randomized study Diabetes Care 2003 26 8 2378 82 10.2337/diacare.26.8.2378 12882865

[B21] Grim PS Gottlieb LJ Boddie A Batson E Hyperbaric oxygen therapy Journal of the American Medical Association 1990 263 16 2216 20 10.1001/jama.263.16.2216 2181162

[B22] Warriner RA III Hopf HW J. J. Feldmeier Enhancement of healing in selected problem wounds Hyperbaric Oxygen 2003: Indications and Results: The Hyperbaric Oxygen Therapy Committee Report 2003 Dunkirk 41 55

[B23] Clark J J. J. Feldmeier Side effects and complications Hyperbaric Oxygen 2003: Indications and Results: The Hyperbaric Oxygen Therapy Committee Report 2007 Dunkirk 137 41

[B24] Londahl M Katzman P Nilsson A Hammarlund C Hyperbaric oxygen therapy facilitates healing of chronic foot ulcers in patients with diabetes Diabetes Care 2010 33 5 998 1003 10.2337/dc09-1754 20427683 PMC2858204

[B25] Zamboni WA Wong HP Stephenson LL Pfeifer MA Evaluation of hyperbaric oxygen for diabetic wounds: a prospective study Undersea Hyperb Med 1997 24 3 175 9 9308140

[B26] Cianci PE Petrone GJ Drager S Lueders HW Salvage of the problem wound and potential amputation wiht wound care and adjunctive hyperbaric oxygen therapy J Hyperb Med 1988 3 127 41

[B27] Oriani G Meazza D Favales F Pizzi G Aldeghi A Faglia E Hyperbaric oxygen therapy in diabetic gangrene J Hyperb Med 1990 5 171 5

[B28] Oriani G Michael M Meazza D Sacchi C Ronzio A Montino O Sala G Campagnoli P Diabetic foot and hyperbaric oxygen therapy: a ten-year experience J Hyperb Med 1992 7 213 21

[B29] Mitton C Hailey D Health technology assessment and policy decisions on hyperbaric oxygen treatment Int J Technol Assess Health Care 1999 15 4 661 70 10645107

[B30] Wang C Lau J New England Medical Center EPC Technology assessments for hyperbaric oxygen therapy for hypoxic wounds and diabetic wounds of the lower extremities (CAG-00060N) 2001 Boston: Agency for Healthcare Research and Quality (AHRQ)

[B31] Agence d'Evaluation des Technologies et des Modes d'Intervention en Sante (AETMIS) Hyperbaric oxygen therapy in Quebec 2000 Montreal: L'Agence

[B32] Medical Services Advisory Committee Hyperbaric oxygen therapy 2001

[B33] Medical Advisory Secretariat Hyperbaric oxygen therapy for non-healing ulcers in diabetes mellitus 2005 Ontario Ministry of Health and Long-Term Care PMC3382405 23074462

[B34] Hailey D Jacobs P Perry DC Chuck A Morrison A Boudreau R Adjunctive Hyperbaric Oxygen Therapy for Diabetic Foot Ulcer: An Economic Analysis 2007 Ottawa: Canadian Agency for Drugs and Technologies in Health

[B35] Kranke P Bennett M Roeckl-Wiedmann I Debus S Hyperbaric oxygen therapy for chronic wounds Cochrane Database Syst Rev 2004 2 CD004123 15106239 10.1002/14651858.CD004123.pub2

[B36] Roeckl-Wiedmann I Bennett M Kranke P Systematic review of hyperbaric oxygen in the management of chronic wounds Br J Surg 2005 92 1 24 32 10.1002/bjs.4863 15635604

[B37] Wang C Schwaitzberg S Berliner E Zarin DA Lau J Hyperbaric oxygen for treating wounds: a systematic review of the literature Arch Surg 2003 138 3 272 9 10.1001/archsurg.138.3.272 12611573

[B38] Wagner FW Jr The dysvascular foot: a system for diagnosis and treatment Foot Ankle 1981 2 2 64 122 7319435 10.1177/107110078100200202

[B39] Borun Center for Gerontological Research Pressure Ulcer Prevention Training Module. Anna and Harry Borun Center for Gerontological Research 2004 http://borun.medsch.ucla.edu/modules/Pressure_ulcer_prevention/pumod.pdf

[B40] Schulz KF Altman DG Moher D CONSORT 2010 statement: updated guidelines for reporting parallel group randomized trials Ann Intern Med 2010 152 11 726 32 20335313 10.7326/0003-4819-152-11-201006010-00232

